# Noninvasive prenatal screening and diagnosis of two fetuses with Williams syndrome in a cohort of 19,607 pregnancies

**DOI:** 10.1080/07853890.2024.2402071

**Published:** 2024-09-12

**Authors:** Weiqiang Liu, Jinshuang Song, Yanmei Zhu, Tong Zhang, Xiaoyi Cong, Xiaojin Luo, Liang Hu

**Affiliations:** Longgang District Maternity & Child Healthcare Hospital of Shenzhen City (Longgang Maternity and Child Institute of Shantou University Medical College), Shenzhen, China

**Keywords:** Williams syndrome, noninvasive prenatal screening, cell-free foetal DNA, enrichment

## Abstract

**Background:**

This study aimed to evaluate the efficiency of noninvasive prenatal screening (NIPS) technology in screening for microdeletions in the 7q11.23 region.

**Methods:**

19,607 pregnant women underwent NIPS in our hospital. Maternal peripheral cell-free foetal DNA (cffDNA) was routinely screened for aneuploidy by cffDNA enrichment and simultaneously analyzed for pathogenic copy number variants (CNVs). The Williams syndrome (WS) 7q11.23 region was targeted in this study. Chromosomal microarray analysis (CMA) was used to verify the screen-positive samples.

**Results:**

The mean concentration of cffDNA before and after enrichment increased from 9.44% to 19.32%, with a statistically significant difference. Two out of 19,607 samples tested for CNVs were found to have a heterozygous deletion at the 7q11.23 region, indicating a high risk for WS. CMA results confirmed the 1.5 megabase (Mb) deletions at the 7q11.23 region in amniotic fluid samples. One of the two WS foetuses had a small left ventricle by ultrasound screening, and the other did not have a significant cardiovascular abnormality phenotype.

**Conclusions:**

NIPS screening for Williams syndrome can be achieved by enriching cell-free foetal DNA and improving bioinformatic analysis algorithms.

## Introduction

Williams syndrome (WS, also known as Williams-Beuren syndrome, WBS, MIM 194050) has a prevalence of 1 in 7500 to 1 in 20000 live births [1, 2]. WS is a neuropsychiatric developmental delay caused by a 1.5 megabase (Mb) to 1.8 Mb microdeletion in the 7q11.23 region [3]. The clinical phenotype of WS is characterized by cardiovascular malformations, distinctive facial features, connective tissue abnormalities, endocrine abnormalities, growth and mental retardation, cognitive difficulties and so on [4]. For the prevention and control of congenital disabilities, early diagnosis of WS is of great value.

The most common cardiovascular abnormalities in patients with WS include supravalvular aortic stenosis (SVAS), peripheral pulmonary artery stenosis (PAS) and mitral valve prolapse (MVP). Less common are aortic insufficiency (AI), ventricular septal defect (VSD), valvular pulmonary stenosis (VPS), aortic arch hypoplasia (AAH) and aortic coarctation (AC) [5]. Prenatally, the significant phenotypes of WS foetuses are intrauterine growth retardation (IUGR, 82.35%), cardiovascular abnormalities (58.82%) including SVAS (40%), VSD (30%), AC (20%), PAS (20%), persistent left superior vena cava (PLSVC, 5.88%), right aortic arch (RAA, 5.88%), cardiac hypertrophy (5.88%). Several other rare abnormalities include echogenic bowel (11.76%), umbilical cord protrusion (5.88%), renal cysts (5.88%), single umbilical artery (SUA, 5.88%), intracardiac highly echogenic foci (IEF, 5. 88%), nuchal dysplasia (5.88%), hydrops fetalis (5.88%) and cord blood deficiency (5.88%) [6].

In theory, prenatal ultrasound can detect these abnormalities, but the diagnosis is difficult in clinical practice [7]. Cardiovascular abnormalities occur in approximately 50% of patients with WS [5, 8], and IUGR is non-specific and may be caused by various maternal, foetal and placental conditions. Therefore, prenatal diagnosis of WS based on prenatal ultrasound is difficult because the prenatal ultrasound features of WS remain incomplete and atypical. To date, only about 90 cases of WS identified by prenatal diagnosis based on ultrasound abnormalities or other prenatal diagnostic findings have been reported [6, 9–13].

In addition to efficient screening for trisomy 21, 18 and 13, noninvasive prenatal screening (NIPS) technology also has good detection efficiency for sex chromosome abnormalities and even copy number variants (CNVs) [14–16]. The screening efficiency of NIPS for pathogenic CNVs (pCNVs) with fragments larger than 6 Mb can reach 83.0%-90.9% [17, 18]. The positive predictive value (PPV) for pCNVs around 3 Mb, such as DiGeorge syndrome, also reached 92.9% [14], and for CNVs size between 2 Mb and 3 Mb, the PPV is about 48.7% [19]. Recently, the American College of Medical Genetics and Genomics (ACMG) recommended that NIPS for 22q11.2 deletion syndrome should be offered to all patients [20], suggesting that whole-genome sequencing-based technology can be achieved for pCNVs with even smaller fragments such as 22q11.2 syndrome.

Direct screening for WS based on NIPS is even less reported. In this study, a Nanosphere sequencing platform was used to perform NIPS screening. Through the enrichment of cell-free foetal DNA (cffDNA) from the peripheral blood of pregnant women and improved bioinformatic analysis algorithms, we found 2 cases of 7q11.23 microdeletion among 19,607 NIPS samples. Prenatal diagnosis confirmed the accuracy of NIPS screening. This study suggests the feasibility of NIPS in screening for WS disease.

## Material and methods

### Subjects

NIPS screening was performed on 19,607 pregnant women who underwent labour and delivery at Shenzhen Longgang District Maternity & Child Healthcare Hospital from November 2022 to August 2023, with ages ranging from 20 to 41 years and gestational weeks ranging from 12 weeks to 27^+6 ^weeks. The study was approved by the Medical Ethics Committee of Shenzhen Longgang District Maternal and Child Health Hospital in accordance with the Declaration of Helsinki’s principles (No. LGFYYXLLQF-2022-003). Written informed consent was obtained from all members before all tests.

### Extraction of cffDNA

The reagents for the extraction, library construction, and sequencing used in this study were provided by the BGI Company (BGI, Wuhan, China). Plasma was isolated by centrifugation of 5 mL whole blood samples at a temperature of 4 °C. In the separated plasma, lysate, proteinase K, and extraction magnetic beads were added to separate DNA from proteins, and then the reagents were eluted to obtain the DNA solution.

### Enrichment of cffDNA

The size of cffDNA fragments is typically small, with most fragments being approximately 160 to 180 base pairs (bp) in length. To remove the small fragments, 39 µL of enrichment magnetic beads were added to the extracted DNA solution, followed by 30 µL of enrichment magnetic beads to remove the large fragments. Then 150 µL of 75% ethanol was added for 2 washes each. Finally, 42 µL of elution buffer was added to elute the DNA, and the supernatant was collected to obtain the enriched DNA solution.

### Library construction and sequencing

An end repair enzyme was added to the enriched DNA to flatten the ends of the fragment. The end-repaired DNA was added to the ligase, buffer and label junction mixture. This allowed each DNA sample to obtain its appropriate molecular identity, followed by purification and polymerase chain reaction (PCR) amplification. The amplified DNA libraries were again purified to remove the residual reagents in the reaction, and then the DNA was eluted to obtain the DNA libraries, each of which was required to be >2ng/μL.

The DNA libraries were mixed proportionally and prepared for sequencing. First, the DNA library was denatured to single-stranded, and then DNA Nanoballs (DNB) polymerase was added to initiate rolling amplification. The concentration was adjusted according to the sequencing requirements. The prepared DNB was then loaded onto the chip for incubation, and the sequencing program was started after the incubation. The sequencer used was BGISEQ-500 from BGI Company.

### Aneuploidy analysis

Raw fastq data were aligned to the human genomic reference sequences (hg19). Low-quality, unaligned, duplicated, and mismatched sequences were filtered out, and sequences uniquely aligned to the genome (unique reads, URs) were retained. Each chromosome on the whole genome was divided into adjacent windows of a specified length, and the number of URs falling in each window and the GC content were calculated. The GC was corrected using the cubic spline interpolation fitting method. The concentration of cffDNA in male foetuses was calculated from the percentage of Y chromosomes, and the concentration of cffDNA in female foetuses was estimated by building a high-dimensional regression model using the nonuniform distribution of cffDNA on the genome. The Z-test was determined for foetal chromosome aneuploidy abnormalities above a specific foetal concentration.

### Algorithms for pathogenic CNV analysis

To filter out pCNVs, we followed the method described in the study by Chen et al. [21]. The human reference genome (hg19) was divided into single-ended 50 bp simulated reads that could be uniquely mapped to the genome. Each chromosome was then divided into adjacent windows of a constant expected number of uniquely simulated reads, with an average window length of approximately 1 Mb.

Raw reads from each sample were aligned to hg19. Low-quality, unaligned, duplicated and mismatched sequences were filtered out, and sequences uniquely aligned to the genome were retained. The number of reads mapped to each window was corrected for the number of reads that would be underrepresented in GC-poor and GC-rich regions during PCR in library preparation and cluster generation. The corrected read number (CRN) mapped to each window was calculated using the Z-score formula.

$$Z_{\text{win}}=\frac{{\text{CRN}}_{\text{win}}-{\text{CRN}}_{\text{mean}}}{{\text{SD}}_{\text{CRN}}}$$

CRN_win_ is the CRN number of a particular window, CRN_mean_ is the mean of the CRN number of that window for all samples in the same lot, and SD_CRN_ is the standard deviation of that window for all samples in the same lot. The Hidden Markov Model (R package HMM,v1.0.1) predicted the Z-score of each window to determine whether it was significantly different, indicating an increase or decrease in CNVs. For the final determination of positive detection, the identified CNVs were compared to a library of known causative CNV diseases (such as the ClinGen database, https://clinicalgenome.org/, and the DECIPHER database, https://www.deciphergenomics.org/).

### Amniotic fluid collection and DNA extraction

5 mL of amniotic fluid was collected by puncture from high-risk pregnant women. According to the instructions, the amniotic fluid sample was used for DNA extraction using the QIAamp DNA Blood Mini Kit (No. 51106, Qiagen, Germany).

### CMA

The Affymetrix Cytoscan 750K chip from the Thermo Fisher Company (Santa Clare, CA, USA) was used. The extracted DNA was digested, ligated, PCR amplified, purified, fragmented and labelled for hybridization according to the instructions, and the washed and stained chip was scanned. Data were collected and analyzed using Chromosome Analysis Suite (ChAS) 4.3 software. Chromosome number was examined for abnormalities at the total level, and copy number variations were examined. CNV results were filtered with a threshold of 100 kilobase pairs (Kb) and 50 probe markers according to the CNV scoring and interpretation recommendation [22].

## Results

### cffDNA enrichment

**Figure 1.**
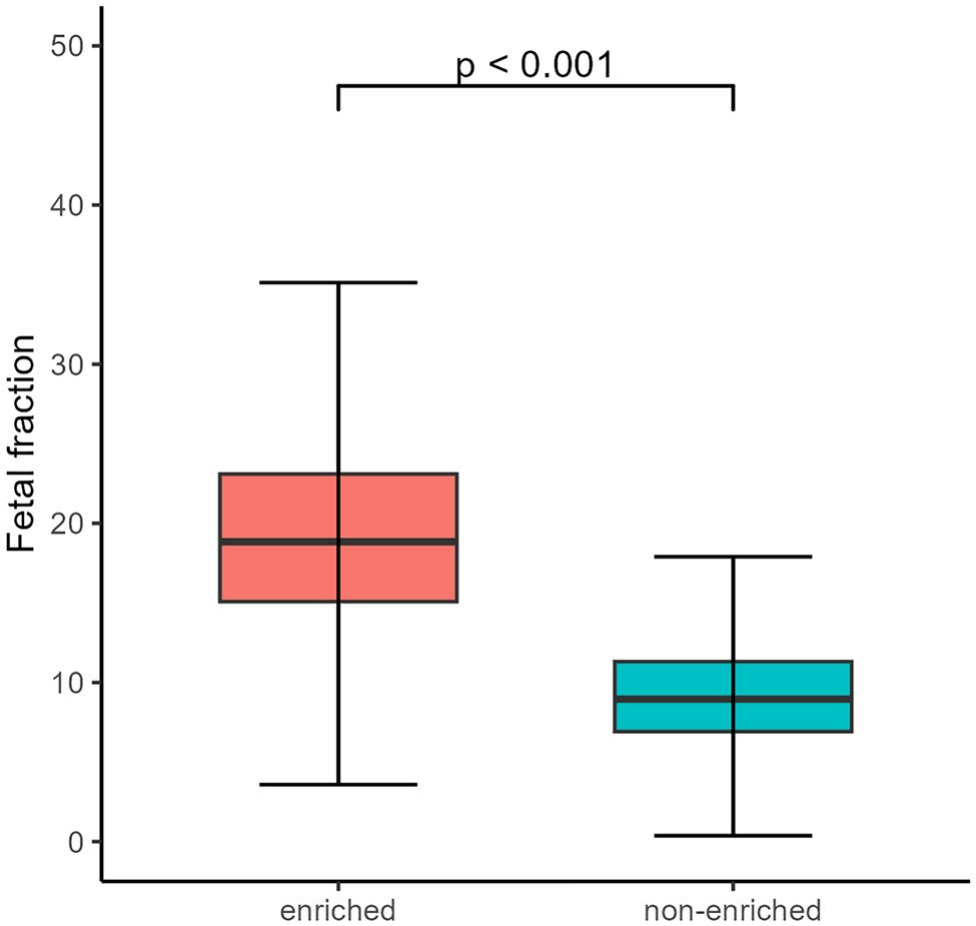
Concentration distribution of cffDNA. CffDNA was enriched after cell-free DNA extraction. As shown in the figure, the average concentration of cffDNA calculated from 19607 samples after applying the enrichment step (left panel) was significantly higher than the concentration of cffDNA derived from 79868 cases without enrichment (right panel), and the difference was statistically significant (p < 0.001) by 2-tailed Student t-test.

Since November 2022, our lab has optimized the NIPS process by adding an enrichment step for the concentration of cffDNA. Before cffDNA enrichment, our lab had a mean cffDNA concentration of 9.44% (3.53% to 28.94%). After the enrichment process, the mean cffDNA concentration was 19.32% (7.57% to 35.54%), and the difference was significant (Table 1 and Figure 1).

**Table 1.** Changes in the concentration before and after the enrichment of cffDNA.

	1^st^*	25^th^*	Median	75^th^*	99^th^*	Mean	SD
Pre-enrichment	3.53%	6.91%	9.58%	11.31%	28.94%	9.44%	4.03%
After enrichment	7.57%	15.08%	18.85%	23.11%	35.54%	19.32%	6.04%

* Percentile; SD: Standard deviation.

### Analysis of pathogenic CNV

**Figure 2.**
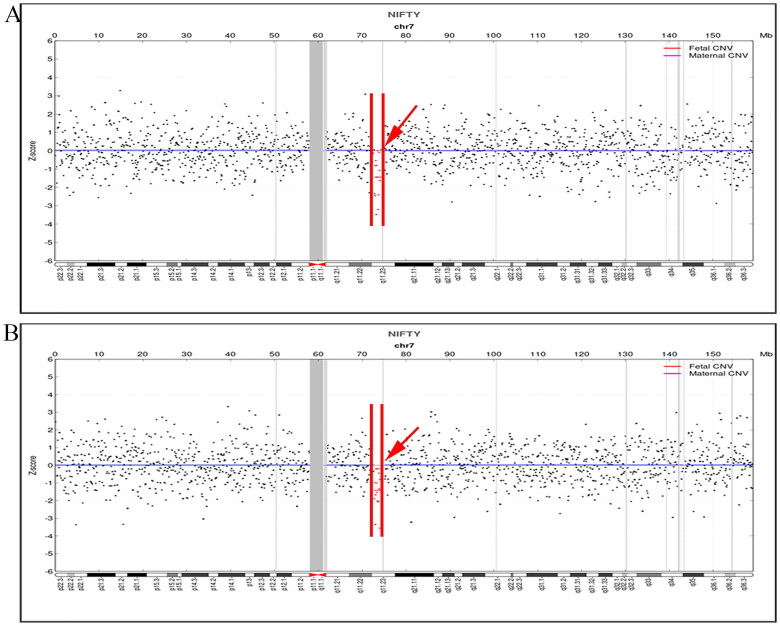
NIPS Indicates the presence of CNV at 7q11.23. (A) Red arrows indicate the presence of a 1.76 Mb microdeletion at 7q11.23 in case 1; (B) Red arrows indicate the presence of a 1.65 Mb microdeletion at 7q11.23 in case 2. The black dots indicate the data points of the NIPS measurement, the red dots indicate the presence of a deletion CNV in the 7q11.23 region, and the red solid line indicates the deletion CNV regions.

Two cases in 19,607 NIPS samples were screened positive for heterozygous 7q11.23 microdeletions, with fragment sizes of 1.76 Mb (Figure 2A) and 1.65 Mb (Figure 2B). The gestational weeks at which the two cases underwent NIPS were 20^+4^ and 14^+5 ^weeks, cffDNA concentrations were 15.00% and 17.70%, and total unique reads were 9.95 million (M) and 5.81 M. The two pregnant women were 36 and 22 years old, respectively.

Other CNVs with clinical significance were also analyzed in this study, such as 22q11 deletion syndrome. Before and after the method improvement, the overall PPV for CNV detection increased from 14.89% to approximately 48.0% (unpublished data).

### Results of CMA

**Figure 3.**
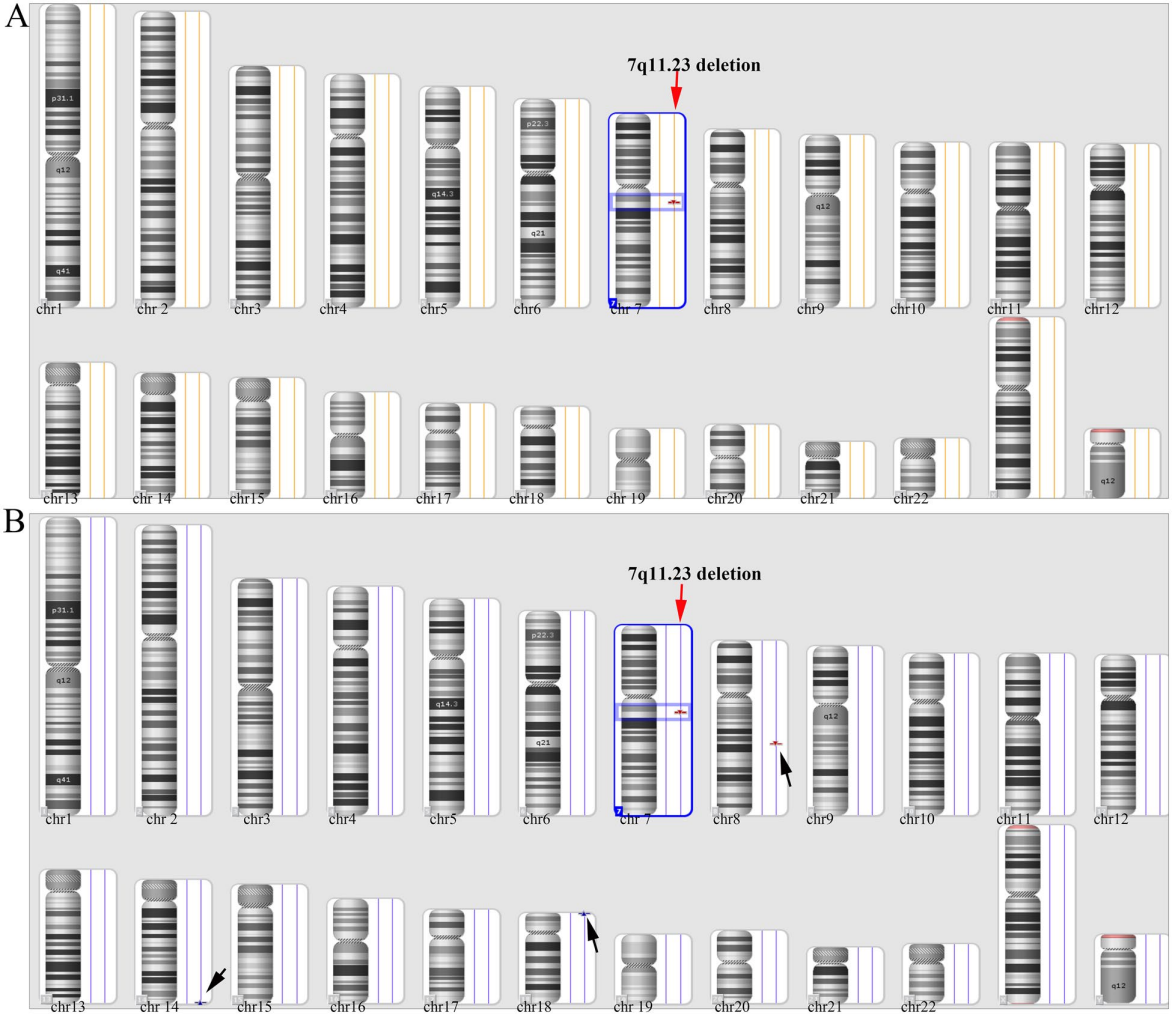
CMA results. (A) CMA results of case 1 showed the presence of a heterozygous microdeletion CNV at 7q11.23 with a fragment size of 1.48 Mb. The specific location of the CNV was arr[GRCh37] 7q11.23(72,669,481_74,154,209)x1(red arrow); (B) a total of four CNVs were detected in case 2, of which 1.49 Mb at 7q11.23 was a pathogenic CNV at arr[GRCh37] 7q11.23 (72,664,089_74,154,209)x1(red arrow) The remaining three were CNVs of uncertain clinical significance or polymorphisms(black arrows). The red triangle on a rectangle indicates a deletion CNV, while the blue triangle indicates a duplication CNV.

At 23^+2^ and 18^+3 ^weeks of gestation, the two pregnant women at high risk for microdeletions at the 7q11.23 region underwent prenatal CMA testing. CMA results confirmed the presence of deletion-type CNV at 7q11.23 in both cases. Data screening revealed that case 1 had only one CNV with a deletion fragment size of 1.48 Mb (Figure 3A). In case 2, four CNVs were detected. A deletion CNV on chromosome 7 with a fragment size of 1.49 Mb at 7q11.23 (Figure 3B), two duplicated CNVs on chromosomes 14 and 18, and another deletion on chromosome 8 were identified, with two CNVs classified as variants of unknown significance and one as a polymorphism (Figure 3B). Analysis of the CNV at 7q11.23 in both samples showed that the deleted region contained 32 genes, including 25 protein-coding genes.

### Ultrasonography

**Figure 4.**
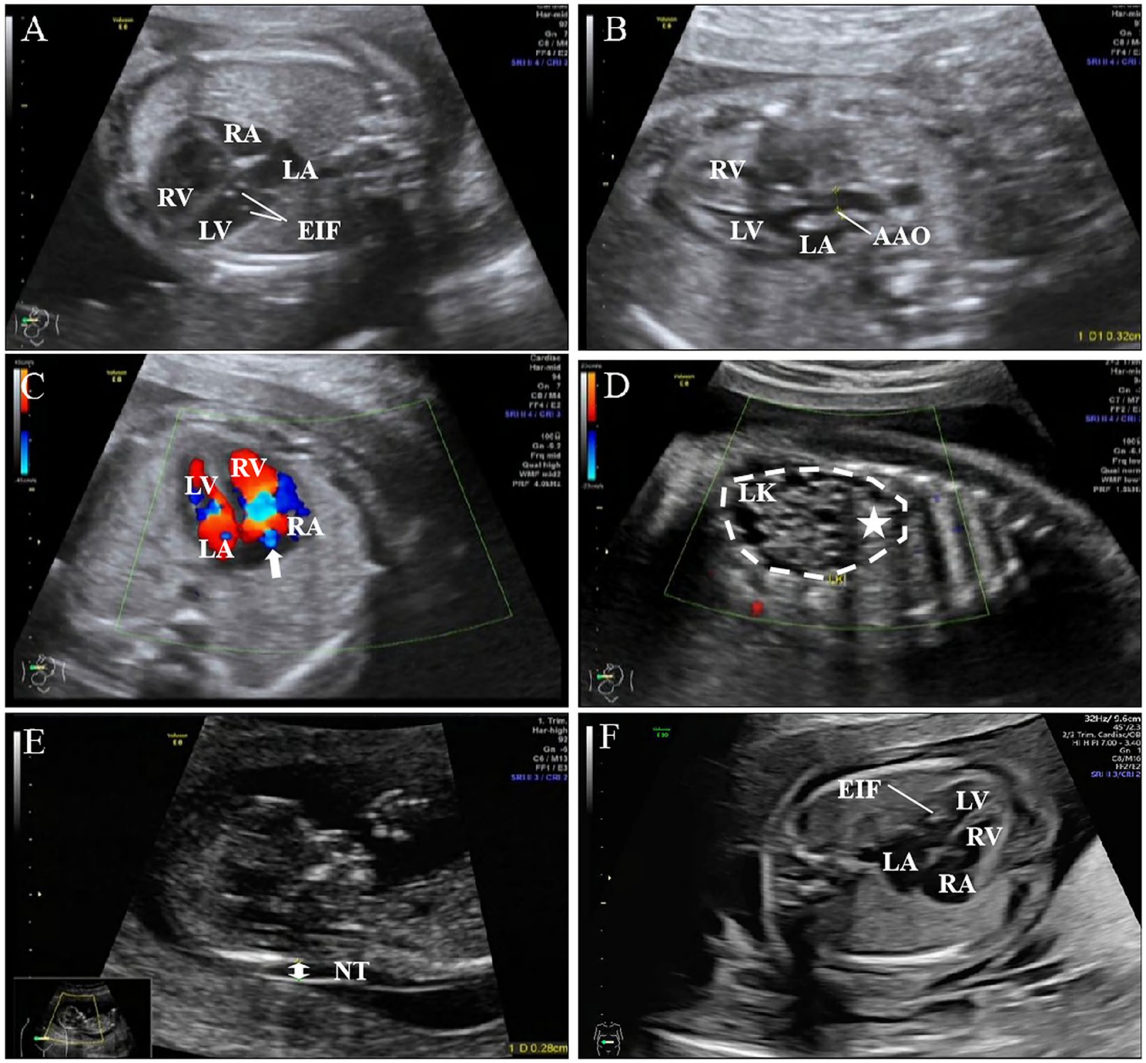
Ultrasound findings in two WS foetuses. Foetus 1 ultrasound showed (A) a left ventricle diastolic transverse diameter of 0.66cm, a right ventricle diastolic transverse diameter of 1.06cm, the left ventricle was significantly smaller than it is shown, the left ventricle was seen in the left ventricle of two echogenic solid points, the diameter about 0.13cm, 0.15cm, (B) the ascending aorta internal diameter of 0.32cm, the ascending aorta internal diameter narrowing; (C) a small amount of tricuspid regurgitation (the white arrow); (D) the left side of the polycystic dysplasia kidney (as indicated by a white star). (E) No significant cardiovascular abnormalities were seen in foetus 2; only a thickening of the NT of 2.8 mm was suggestive (double-sided arrow), and (F) an echogenic spot was seen in the left ventricle of foetus 2. Abbreviation: LA: left atrium; LV: left ventricle; RA: right atrium; RV: right ventricle; EIF: Echogenic intracardiac focus; AAO: Aorta ascendens; LK: Left kidney; NT: Nuchal translucency

Two WS foetuses underwent ultrasound examination. In one case, the foetal ultrasonography showed a slightly smaller left heart with a small amount of tricuspid regurgitation, combined with sonographic changes in the left kidney, suggesting a left polycystic dysplastic kidney (Figure 4A-D). The other foetal ultrasound showed no cardiovascular abnormalities (Figure 4E, F).

## Discussion

WS is a severe congenital disorder characterized by developmental delay, mild to moderate intellectual disability, specific cognitive problems, cardiovascular disease (supravalvular aortic stenosis, peripheral pulmonary stenosis, hypertension), connective tissue abnormalities, endocrine abnormalities (precocious puberty, hypercalcemia, hypercalciuria, hypothyroidism), and a distinctive facial appearance [3]. A deletion in the chromosomal region 7q11.23 of approximately 1.55 Mb to 1.83 Mb, encompassing 32 genes, including the elastin gene (*ELN*), is the primary cause of the disease [23]. WS is recommended as one of the 121 severe rare genetic diseases for diagnosis and treatment in China.

Studies have shown that pCNVs at the genomic level have a carrier rate of up to 1/270 in the population [24]. The percentage of foetuses with clinically significant CNV in pregnant women can range from 1.6% to 1.7% [25], regardless of maternal age and gestational week. Therefore, NIPS is an essential tool for the early detection and diagnosis of these conditions.

NIPS technology has been introduced into clinical practice to screen for genomic disorders or pCNVs. The ACMG and the International Society for Prenatal Diagnosis (ISPD) have issued guidelines or statements indicating that screening for specific pCNVs with clear clinical significance and severe phenotypes is feasible, provided informed consent is obtained. The detection rate, specificity, PPV, negative predictive value (NPV), and other indicators of the CNVs to be detected are clarified [26, 27]. The effectiveness of NIPS screening for CNVs has improved dramatically with the enrichment of cffDNA concentration, increased sequencing depth, and further optimization of the bioinformatic analysis process. With 10 million to 19 million validated sequencing data, the PPV for 22q11.2 microdeletion syndrome was up to 93%, for Prader-Willi/Angelman syndrome up to 75%, and for Cri du Chat syndrome up to 50% [14], suggesting that NIPS screening for pCNVs is feasible.

Most current studies on the fragment size of pCNVs have chosen 3 Mb to 5 Mb as the sensitive detection limit for NIPS screening of pCNVs [28–30]. For smaller CNVs, extensive data are still needed to support the screening efficacy of NIPS. The sensitivity and specificity of NIPS screening for pCNVs larger than 1 Mb and smaller than 5 Mb can be as high as 83.33% and 99.34%, respectively. However, the literature has not reported the early screening of WS disease by NIPS. Therefore, this study attempted to screen for WS disease by improving the NIPS technique and algorithm.

The length of the CNV fragment, the amount of validated data for NIPS sequencing, and the cffDNA concentration are the main factors affecting the efficiency of NIPS screening for pCNVs. CNVs in WS are 1.5 Mb-1.8 Mb, such small CNVs are currently not used for routine screening by NIPS. In this study, the average cffDNA concentration before the implementation of cffDNA concentration enrichment was 9.44%, and after the implementation of cffDNA concentration enrichment, the average cffDNA concentration increased to 19.32%. Foetal cffDNA enrichment effectively reduces the false-positive [31] and false-negative aneuploidy rates [32].

Although the detection of WS by NIPS has also been reported in the literature, in contrast to the present study, the samples were subjected to NIPS retrospectively in the presence of a definitive prenatal diagnosis [33]. Nevertheless, this confirms that NIPS can be effective in the detection of WS. The literature has also reported that NIPS did not detect seven cases of WS using the BGI platform [6], suggesting that applying NIPS technology for WS screening still requires caution and that stable and reliable conditions need to be established.

In this study, we effectively increased the detection efficiency of CNVs by cffDNA enrichment without increasing the corresponding sequencing volume. Using the Nanosphere sequencing technology platform, with an average of approximately 10 million unique reads per sample in our laboratory and an approximately twofold increase in cffDNA concentration, two WS foetuses out of nearly 20,000 samples were detected by cffDNA enrichment, which is consistent with the prevalence of WS. Retrospective analysis of data from 2017 to before 2022 did not exclude the possibility of underdetection by NIPS based on the prevalence estimate of WS, as the laboratory did not perform foetal concentration enrichment at that time, and no cases of WS were detected in nearly 80,000 samples.

With the improvement of CNV algorithms and cffDNA concentration enrichment, we successfully screened out two WS foetuses. In this study, we analyzed CNVs at 7q11.23 in WS, and identified rare CNVs, such as cases with 22q11 syndrome, using this improved method. The PPV for CNV detection increased from 14.89% [34] to 48.0% in our laboratory (unpublished data), further suggesting the efficiency of this improvement method. However, there is a limitation to this study: only two positive WS cases were identified, and more samples were needed to confirm the sensitivity and specificity of our NIPS-based WS screening method further. Nevertheless, our study suggests that noninvasive screening techniques can achieve early screening and diagnosis of WS, allowing early diagnosis and effective intervention for WS.

## Conclusion

In conclusion, this is the first report on WS that was directly screened by NIPS technology. In this study, NIPS screened positive two cases of WS foetuses that did not have a typical phenotype and was followed by CMA validation, suggesting that NIPS screening for Williams syndrome can be achieved by enrichment of cell-free foetal DNA and improvement of bioinformatic analysis algorithms.

## Data Availability

The data that support the findings of this study are available on request from the corresponding author, [WQ.L]. The raw data are not publicly available due to their containing information that could compromise the privacy of research participants.
